# Use of machine learning techniques to identify HIV predictors for screening in sub-Saharan Africa

**DOI:** 10.1186/s12874-021-01346-2

**Published:** 2021-07-31

**Authors:** Charles K. Mutai, Patrick E. McSharry, Innocent Ngaruye, Edouard Musabanganji

**Affiliations:** 1 African Center of Excellence in Data Science, University of Rwanda, Kigali, BP 4285 Rwanda; 2 College of Engineering, Carnegie Mellon University Africa, Kigali, BP 6150 Rwanda; 3 Oxford Man Institute of Quantitative Finance, Oxford University, Oxford, OX2 6ED UK; 4 College of Science and Technology, University of Rwanda, Kigali, Rwanda; 5 College of Business and Economics, University of Rwanda, Kigali, Rwanda

**Keywords:** Socio-behavioral, Screening, High-risk, Predictors, XGBoost

## Abstract

**Aim:**

HIV prevention measures in sub-Saharan Africa are still short of attaining the UNAIDS 90–90-90 fast track targets set in 2014. Identifying predictors for HIV status may facilitate targeted screening interventions that improve health care. We aimed at identifying HIV predictors as well as predicting persons at high risk of the infection.

**Method:**

We applied machine learning approaches for building models using population-based HIV Impact Assessment (PHIA) data for 41,939 male and 45,105 female respondents with 30 and 40 variables respectively from four countries in sub-Saharan countries. We trained and validated the algorithms on 80% of the data and tested on the remaining 20% where we rotated around the left-out country. An algorithm with the best mean f1 score was retained and trained on the most predictive variables. We used the model to identify people living with HIV and individuals with a higher likelihood of contracting the disease.

**Results:**

Application of XGBoost algorithm appeared to significantly improve identification of HIV positivity over the other five algorithms by f1 scoring mean of 90% and 92% for males and females respectively. Amongst the eight most predictor features in both sexes were: age, relationship with family head, the highest level of education, highest grade at that school level, work for payment, avoiding pregnancy, age at the first experience of sex, and wealth quintile. Model performance using these variables increased significantly compared to having all the variables included. We identified five males and 19 females individuals that would require testing to find one HIV positive individual. We also predicted that 4·14% of males and 10.81% of females are at high risk of infection.

**Conclusion:**

Our findings provide a potential use of the XGBoost algorithm with socio-behavioural-driven data at substantially identifying HIV predictors and predicting individuals at high risk of infection for targeted screening.

**Supplementary Information:**

The online version contains supplementary material available at 10.1186/s12874-021-01346-2.

## Background literature

HIV continues to be significantly the most threatening infectious disease and a burden to public health globally. In the year 2019, global estimates show that 38 million people are living with HIV while 1.7 million and 690,000 thousand are reported new cases and deaths respectively, despite the remarkable progress in diagnosis and access to antiretroviral therapy (ART) [1]. More than half of people living with HIV, 42.9% of new infections, and 43.5% of deaths due to AIDS are concentrated in East and Southern Africa [1]. In 2018, 1.6 million, 1 million, 210,000 thousands and 1.2 million people were living with HIV, 72,000, 38,000, 7,800 and 48,000 were newly infected people and 24,000, 13,000, 2,400 and 17,000 deaths were from AIDS-related illness in Tanzania, Malawi, Eswatini and Zambia respectively [2]. The Joint United Nations Programme (UNAIDS) had set goals towards stopping AIDS as a public health threat by 2030 [3, 4]. However, the COVID-19 pandemic is already thwarting the progress made, and it can adversely lead to additional AIDS-related deaths in sub-Saharan Africa by the end of 2021 [5, 6].

Despite universal HIV intervention efforts in East and Southern Africa, the geographical distribution of the HIV epidemic is still widely varied [7, 8]. The region being a resource constraint can not have every intervention for everyone and everywhere. Granular information concerning the HIV epidemic needs tailor-made solutions to address and help protect specific individuals [9]. To identify the most vulnerable individuals for the infection globally, strategies are geared towards optimal allocation of resources and thus higher impact and efficiency contrary to a homogeneous distribution of resources [10, 11]. Behavioural and social-demographic factors are among significant contributions of HIV transmission and require investigation on the nature of the impact on the HIV epidemic in a particular population [12]. Despite HIV screening being an effective method of identifying individual status, it has challenges and constraints [13]. Community-based HIV screening has successfully improved the identification of people living with HIV [14]. One of the ways of diagnosing people living with HIV is through universal screening of individuals attending health care facilities, but this can be costly for the low-risk population compared to selective testing of those at high risk [15]. Including social-demographic factors in the analysis may extensively improve the potential of predicting those at higher risk of the infection, enhancing optimal choices in the screening process, and helping to facilitate testing and counselling for HIV [16]. This may also disclose individuals who may need PrEP, among other necessary early interventions [17, 18].

Machine learning entails the utilisation of computational and statistical algorithms to determine hidden associations of data that might increase predictions through relaxation of the modelling postulates advanced by standard approaches [19]. Among the recent advances in prediction tools and identification techniques in HIV statistical data [20–22], machine learning offers greater capability in processing huge amounts of data. Its recent application in the identification of potential candidates for preexposure prophylaxis (PrEP) in the USA and Denmark and a population-based research setting in Eastern Africa highlights some of its capabilities [23]. Klon et al. used Laplacian-modified naïve Bayesian to identify active inhibitor compounds from a target database [24]. Another example is the use of electronic health record data in developing HIV prediction models for identifying PrEP candidates in an extensive healthcare system [25]. A machine learning algorithm has been developed that can automatically select important HIV risk-related variables using patients' demographic and clinical data [26].

A review of the use of machine learning approaches in studying HIV/AIDS infection was previously published [27]. The paper by Lee et al. used machine learning approaches in classifying patients with and without the toxicity of biomarkers of mitochondrial in HIV [28]. Recently, Orel et al. used machine learning techniques on the Demographic Health Survey of 10 countries to identify HIV Positive individuals [29].

This paper aims at using machine learning algorithms to identify the HIV predictors of persons using socio-behavioural features and predict those at increased risk of infection in the East and Southern African countries.

## Methods

### Data

We used the Population-based HIV Impact Assessment (PHIA) project that consists of cross-sectional household-based surveys designed to assess HIV-related key health indicators [30]. ICAP, based in Columbia University in collaboration with the US Centers for Disease Control and Prevention (CDC) and the ministries of Health, manages and implements the PHIA project. The PHIA project is assessing programs of HIV in countries supported by the President's Emergency Plan for AIDS Relief (PEPFAR) by national household surveys.

It was established in 2015 and geared towards documenting the achievements of HIV programs in participating countries as well as ensuring a better understanding of the regional burden trends of the disease in developing countries. PHIA conducts surveys in 14 countries: Côte d’Ivoire, Cameroon, Ethiopia, Eswatini, Haiti, Kenya, Lesotho, Zimbabwe, Malawi, Namibia, Rwanda, Tanzania, Uganda and Zambia. More details on the PHIA survey have been reported elsewhere [31].

We only included individuals tested for HIV in our analysis from the recently released PHIA survey data for Tanzania (2016–2017), Zambia (2016), Malawi (2015–2016) and Eswatini (2016–2017). Countries whose data were not yet released were excluded from the study. We merged adult datasets and HIV test results from the four countries to obtain two sets of data, comprising 41,939 male and 45,105 female respondents with 8.5% and 13.3% HIV positive cases respectively, Table 1. Background characteristics of the dataset are displayed in table A3. We considered two HIV test outcomes for respondents, positive and negative, thereby requiring the construction of a binary classifier using machine learning.

**Table 1:** Summary of Phia dataset

Characteristics	Levels	Overall	HIV Positive	HIV Negative
n (Total number of individuals,%)		87,044	9,533 (11.0)	77,511 (89.0)
Gender, n (%)	Males	41,939	3,552 (8.5)	38,387 (91.5)
	Females	45,105	5,981 (13.3)	39,124 (86.7)
Country, n (%)	Malawi	19,829	2,100 (10.6)	17,729 (89.4)
	Eswatini	11,875	3,230 (27.2)	8,645 (72.8)
	Zambia	21,280	2,569 (12.1)	18,711 (87.9)
	Tanzania	34,060	1,634 (4.8)	32,426 (95.2)

### Data pre-processing

**Fig. 1:**
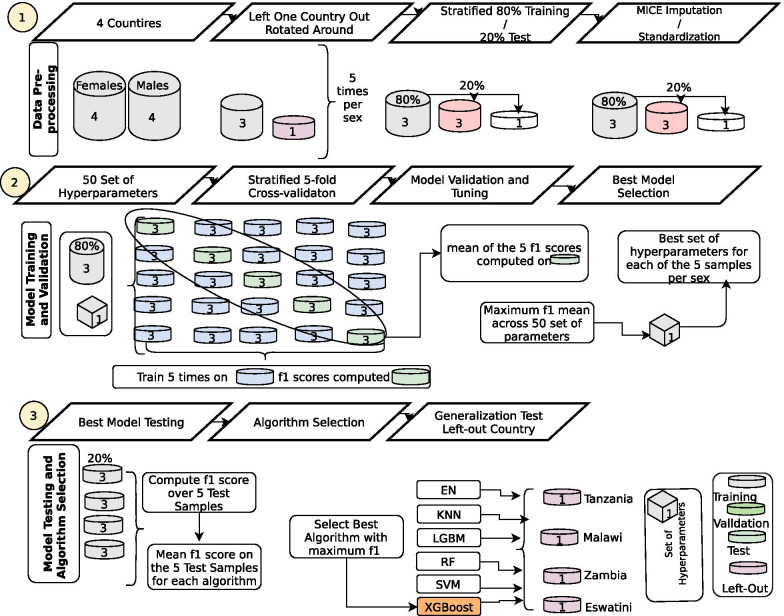
Diagram explaining the method process

We pooled datasets from the four countries and merged HIV test results with the adult interview datasets. We then resampled the data utilising sample weights of HIV test outcomes per country thus compensating for non-coverage, non-response and population total adjustment weights. Then, we joined the data sets from the four countries into one data frame with 238 variables each for both sexes. We removed variables with more than 30% missing values, those with no variance, non-unique columns, above 80% correlated features, indeterminate and non-informative features such as household-id, person-id, line-number and others, Table A1. We also encoded both the nominal and ordinal variables using the label-code and one-hot encode methods appropriately based on the information from the survey [32]. Multiple imputations with chained equations (MICE) [33] was utilized in imputing the missing values in each of these categories. Finally, we further harmonised and scaled the data by standardizing to ensure a fair penalisation of the scheme used for all the regressors, Fig. 1, step 1. This resulted in 41,939 males and 45,105 females in the final dataset corresponding to 26 and 36 variables respectively as shown in, Table A2. From this final dataset, 25 variables of the total variables were similar for both sexes.

### Model validation

In this study, our machine learning task was structured to solve a binary classification problem.

Our dataset comprises healthy individuals labelled negative in one class while the infected individuals are labelled positive in the other class.

We left out one country for later testing and this was rotated around for testing the generalisation of the models separately for males and females. 80% of the datasets were selected for training while 20% were used as test samples, Fig. 1, step 1. We chose an 80:20 ratio for our study and it has been shown to achieve the best results among other ratios elsewhere [34]. We randomly picked from a grid 50 sets of control values of the learning process (hyperparameters), and these were used in training and validation of data using each of Elastic Net (EN) [35], k-Nearest Neighbors (KNN) [36], RandomForest (RF) [37], Support Vector Machine (SVM) [38], XGBoost [39] and Light Gradient Boosting (LGBT) [40] algorithms, (Fig. 1, step 2 and 3).

We determined the average scores of f1 for each of these 50 sets with a five-fold cross-validation plan over the validated samples and the most powerful set of hyperparameters were picked, Fig. 1, step 2. f1 score is a metric that is the most-used member of the parametric family of the f-measures, named after the parameter value β = 1, where beta is a factor of recall importance than precision [41]. It is defined as the harmonic mean of precision and recall.

$$\mathrm f1\;\mathrm{score}\:=\:2\ast(\mathrm{Recall}\;\ast\;\mathrm{Precision})\;/\;(\mathrm{Recall}\:+\:\mathrm{Precision})$$

Precision is the ratio of correctly predicted positive observations to the total predicted positive observations and recall (sensitivity) is the ratio of correctly predicted positive observations to all observations in the actual class. It accounts for both false positives and false negatives and it cannot be influenced by the uneven class distribution and therefore preferred over the accuracy metric [42]. Importantly, the metric used was very sensitive and high yielding in predicting the number of HIV positive persons (precision). We computed the Precision-Recall for our preferred model per sex, displaying the precision for different sensitivities. This curve is not affected by imbalanced datasets and hence preferred over the ROC curve [43].

### Model and variable selection with the direction of the association

The algorithm with the best f1 score was used in the subsequent analysis where all **c**ountries were included. We used the algorithm along with 250 sets of parameters random search in training and validation with a five-fold cross-validation plan. First, we estimated and compared sensitivity, f1 score and positive predictive value results on all the variables. We then conducted a sequential forward floating selection (SFFS) procedure in determining the saturation point of variables based on f1 scoring with 80% training samples and variables whose f1 scores plateaued from the saturated point were selected. We also evaluated the association of the selected features with the probability of being HIV positive using SHapley Additive exPlanations (SHAP) [44].

SHAP is an important technique that is used in explaining the contribution of each feature in prediction.

To determine a 95% sensitivity of individuals living with HIV knowing their status and a 95% or more probability of individuals being HIV positive we utilized our preferred model to calibrate the given two situations. First, a 95% sensitivity was set equivalent to 95% of persons knowing their status and chose a threshold that corresponds to this sensitivity, reporting precision and number of individuals to be tested. Secondly, a population for which the predicted probability of being HIV positive was 95% or higher was identified. These individuals were considered as either positive targets for testing strategies or HIV negative individuals for prevention aid.

## Results

Overall, there was a varied HIV prevalence ranging from 4·8% in Tanzania to 27·3% in Eswatini. The overall HIV positivity was 8·5% for male and 13·3% for females, Table 1. Persons aged 35 years and above displayed a higher HIV prevalence than the younger population in both sexes. In urban regions, 51.1% of females have HIV, compared to 48.8% who do not. Similarly, 49.2% of males in urban areas have HIV, compared to 47.0% who do not. In the wealth quintiles of males, 32.3% of the more wealthy persons have HIV, compared to 30.75% of those who do not. In the wealth quintiles of females, 29.8% of more wealthy individuals have HIV, compared to 30.9% of those who do not. 51.0% of males with the lowest level of education have HIV, compared to 549% who do not. Again, 61.4% of females with the lowest education level have HIV, compared to 59.9% who do not, Table A3.

### Algorithms results in test samples

**Fig. 2:**
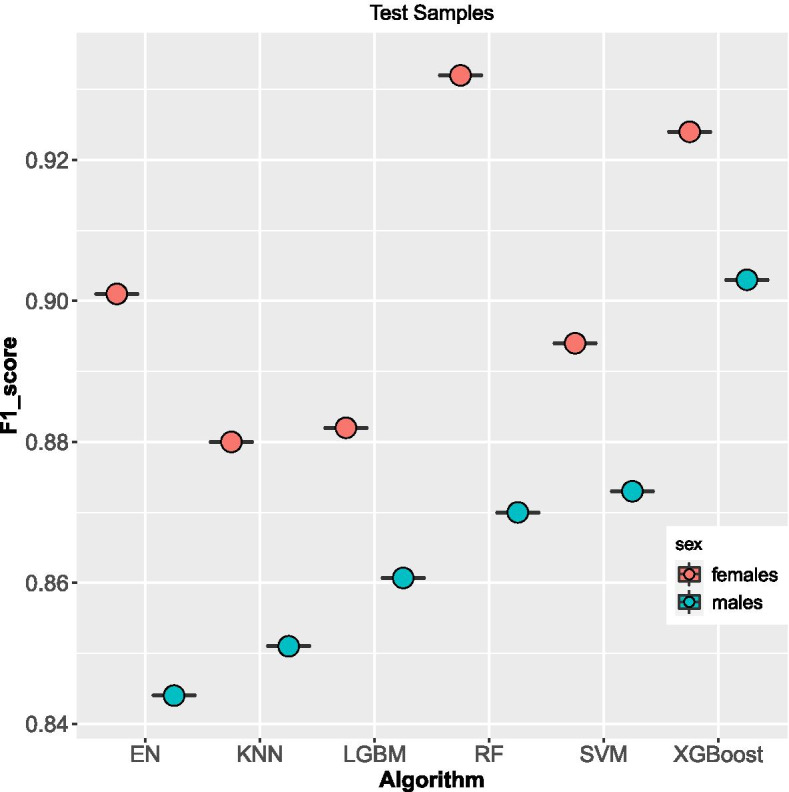
f1 scores Boxplot on methods used on test samples per sex

Figure 2, shows that the XGBoost algorithm achieved the highest f1 score of 90% and 92% for males and females respectively, among the six algorithms that were used on all 8 (4 per sex) test samples. This was followed closely by the RF algorithm with a score of 87% for males and 93% for females. EN algorithm performance was 84% and 90% for males and females respectively. SVM performance was 87% for males and 89% for females. The LGBM f1 score was 86% for males and 88% for females while KNN performed dismally with an f1 score of 85% for males and 88% for females, Table 2.

**Table 2:** F1 score for Algorithms on the test, left-out and train samples

samples	XGBoost	KNN	SVM	RF	EN	LGBM
males test	0.90	0.85	0.87	0.87	0.84	0.86
females test	0.92	0.88	0.89	0.89	0.90	0.88
males left-out	0.83	0.81	0.79	0.79	0.72	0.81
females left-out	0.85	0.85	0.86	0.86	0.76	0.87
males train	0.90	0.85	0.86	0.91	0.83	0.86
females train	0.91	0.87	0.89	0.92	0.88	0.88

### Algorithms results in left-out samples

**Fig. 3:**
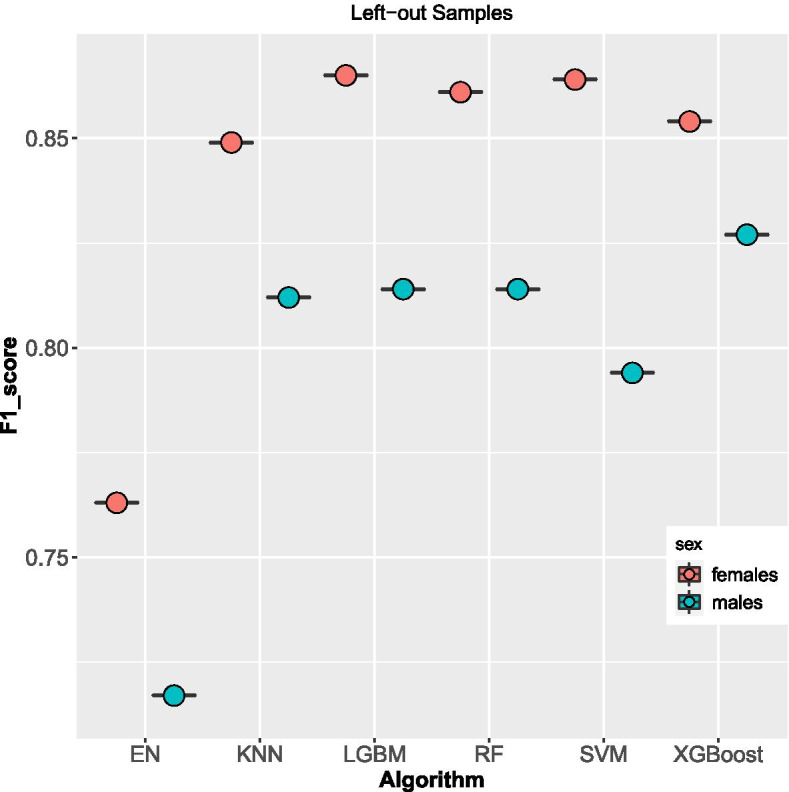
f1 scores Boxplot on methods used on left-out samples per sex

Similarly, the six algorithms were trained on all four left-out samples. The f1 scores between males and females substantially varied all the algorithms, Fig. 3. However, the XGBoost algorithm got the highest f1 score of 83% and 85% for males and females respectively, among the six algorithms. This was followed closely by the LGBM algorithm with a score of 81% for males and 87% for females. SVM algorithm performance was 79% and 86% for males and females respectively. KNN secured a score of 85% for females and a low score of 81% for males. RF scored 86% for females and 81% for males while EN was the worst-performing algorithm with an f1 score of 76% for females and 72% for males, Table 2.

### Variable selection and direction of associations

**Fig. 4:**
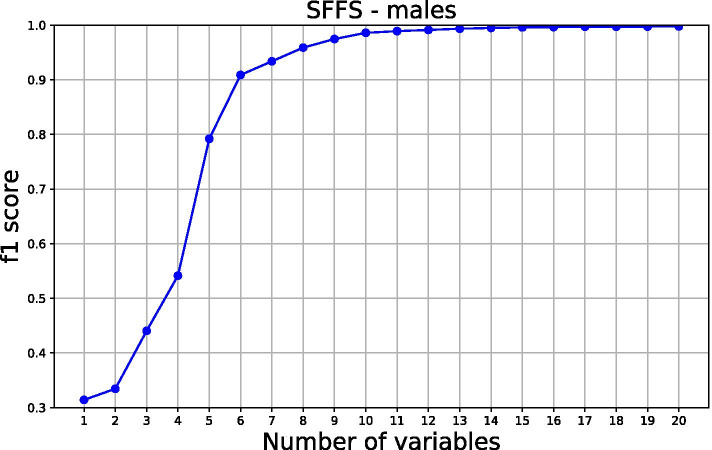
Sequential floating forward selection (SFFS) for males

**Fig. 5:**
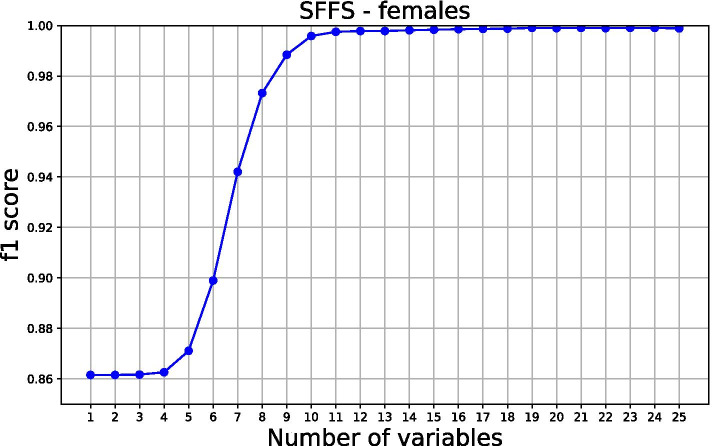
Sequential floating forward selection (SFFS) for females

**Fig. 6:**
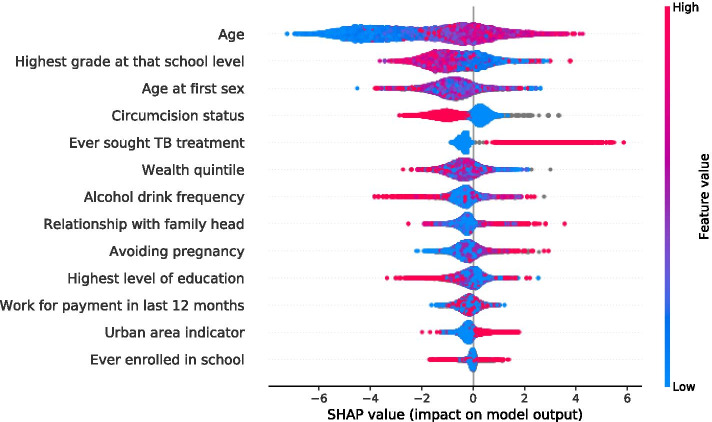
SHAP summary plots for HIV status predictors in male individuals

**Fig. 7:**
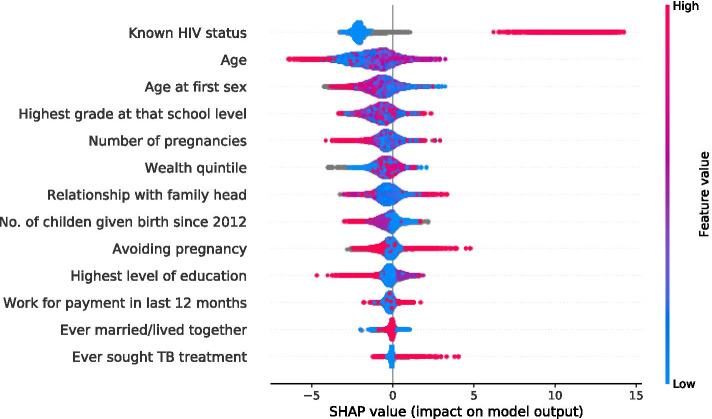
SHAP summary plots for HIV status predictors in female individuals

SFFS procedure was used in determining the saturation limit, selecting variables based on f1 scoring. As a result, 15 and 12 most influential features of males and females were selected respectively, Fig. 4 and 5. To understand how a feature contributes to the output of the model, we plot SHAP values, Fig. 6 and 7 for males and females respectively. These variables are displayed after ranking in descending order, bearing the highest average or median values of Shapley at the top.

Here, all the values on the left represent the observations that shift the predicted value in the negative direction while the points on the right contribute to shifting the prediction in a positive direction. The graph summarises the impact of explanatory features on the model output. Features that increase or decrease the risk of HIV infection are coded in red and blue respectively. Being older, never attending school, at the highest level of education, at the highest grade a school level, in avoidance of pregnancy, in TB treatment, in use alcohol drink, an urban dweller, aware of HIV status, wealthy, nonmarital and circumcised is predictive of HIV positivity.

To determine a 95% sensitivity of individuals living with HIV knowing their status and a 95% or more probability of individuals being HIV positive we utilized the XGBoost model to calibrate the given situations A and B respectively.

### Situations

#### 95% of individuals living with HIV know their state

Table 3, shows confusion matrices on test samples. A sensitivity of 95% for males would need 4,154 individuals out of 8,388 (49.52%) tested to identify 651 HIV positives from 685 persons living with HIV. Correspondingly, therefore, five individuals would need testing to find one person who is HIV positive with a precision of 15.67%. 3,301 individuals out of 9,021 (36.59%) of females would require testing to detect 1,115 HIV positives out of the 1,173 persons living with HIV. Similarly, the precision is 33.77% and needs 19 individuals tested.

**Table 3:** People living with HIV know their status and 95% or more probability of being HIV positive

95% of those with HIV	TP	FP	FN	TN	precision
Know their status (males)	4200	3503	34	651	15.67
Know their status (females)	5662	2186	58	1115	33.77
95% or > probability of	7690	13	350	335	99.26
being HIV positive (males)					
95% or more probability of	7842	6	204	969	96.26
being HIV positive (females)					

#### 95% or higher probability of being HIV positive

We identified 348 (4·14%) males out of the 8,388 and 975 (10.81%) females out of 9,021 as a high-risk population. We find that 350 males would have been identified HIV positives out of 685 people living with HIV while 969 females would have been correctly identified HIV positives from 1,173 individuals, Table 3.

## Discussion

We used a large dataset of over 80,000 respondents in four countries from the East and Southern Africa region to predict the HIV status of persons by use of socio-demographic factors. We used the XGBoost method in the identification of the most predictive factors of HIV positivity, which delivered better results than the other five algorithms with f1 scores on the sample test of 90% and 92% when all variables are included in males and females respectively.

The method enabled us to establish the most predictive features for HIV status in both sexes: age, relationship with the head of the family, ever enrolled in school, the highest level of education, highest grade at that school level, work for payment in the last 12 months, wether avoiding pregnancy, age at first sex experienced, ever sought TB treatment, frequency of alcohol drinking, urban area indicator, wealth quintile, number of pregnancies, number of births since 2012, marital status and circumcision status.

The course of the association between predictor features and HIV status of individuals was determined through the use of XGBoost along with SHAP plots and illustrated specific feature importance to give an intuitive understanding of the key features. The age of an individual has the highest overall impact on HIV status than other features, and any change of age can have a more remarkable influence than others. More aged individuals have a higher probability of infection in both sexes. Several of those avoiding pregnancy by various methods stand a higher chance of contracting the disease in both sexes. Potential reasons for this may be an increased exposure to sex making them more vulnerable. The majority of those living in urban regions seemed to be more exposed to the disease than their counterparts living in rural areas. Those with a little level of education have low knowledge of mitigation measures of HIV risk and pose a greater risk of HIV in both sexes. HIV positivity is associated with a higher number of those seeking TB treatment; a low CD4 count from HIV patients are at a much higher risk of falling ill from TB infection than those who are negative. Similarly being uncircumcised exposes males to the disease which is consistent with studies by [45] while in females alone, being exposed to sex at an older age, attaining higher grade at school, an increase in the number of children born may lead to a reduction of HIV positivity. Our results are consistent with those of [46] that indicated literacy and urbanity as strong predictors of HIV acquisition and Sing et al., which found that urban dwellers may increase HIV positivity through more contact with high-risk sexual individuals than rural residents [47]. Age, a little level of education and gender being predictors of the disease assert the findings in [48, 49].

A 95% sensitivity was required in ensuring that 95% of individuals living with HIV knew their status. With the XGBoost algorithm, we utilized 15 and 12 most predictive variables of males and females accordingly to establish 5 and 19, the number required to screen to know one individual with HIV in males and females respectively. These are within the range of 3 to 86 and 4 to 154 for community-based and facility-based screening respectively given in previous studies [50]. We identified 4·14% males and 10.81% females as a high-risk population in the second situation, which is consistent with previous studies that indicated that about seven women get new infections with HIV for every four men infected [51, 52]. In general, female performance in all our algorithms was slightly higher than those of males in this study. Our method borrows heavily from Orel's approach in predictor identification, and they both choose the Xgboost model algorithm as the best performing model among the alternatives. Our results, on the other hand, show different predictors than those found in Orel's research, with the exception that they both found individual age and wealth as predictors of the disease [29].

Other screening methods exist, but they are not without drawbacks. Universal screening, in which patients are given tests on health care facilities, is limited to a poor cost–benefit ratio in low HIV incidence situations [53]. While indicator-condition-guided testing based on specific medical conditions ignores factors such as age, sex, and medical conditions, which have all been linked to a lower risk of HIV transmission [54]. In generalized epidemic settings, a focus on well-known risk groups such as serodiscordant spouses and young women can effectively reach high-risk individuals [55] but may overlook less well-known or easily defined subgroups at risk [56], resulting in inefficient resource allocation [57]. Despite the absence of a recognized risk factor, self-assessment is one method of recognizing individuals at high risk. However, an individual's risk perception is influenced by their HIV-related awareness, and unanticipated or uncontrolled exposures can go undetected [58]. According to WHO guidelines, PrEP should be targeted at subpopulations considered to be at high risk of HIV infection [59]. However, in the context of a widespread outbreak, the best demographic subgroups to target may not be obvious, and merely providing PrEP to established high-risk subgroups like young people or mobile populations may be ineffective. As a result, a PrEP technique based on more subtle use of individual characteristics may be able to reduce the cost of preventing a new HIV infection. Our method provides an alternative to some of the drawbacks listed above, as well as a potential complementary method for identifying people who may gain most from enhanced mitigation strategies. One limitation of this study is the validity of our model. There was a high degree of missingness and inconclusiveness from self-reported data that potentially impacted the training data.

Our conclusion might reveal the social-behavioural identification of HIV and can enhance screening approaches in limited resources situations. There is a need to adapt HIV screening strategies that better target the adult population, those using contraceptives, urban dwellers, the little educated population, TB patients and uncircumcised men. There is an increased number of available surveys with individual-level data that is rich in demographic characteristics, social history, laboratory tests and results of various diseases. More advanced approaches to utilize them can effectively assist in preventing, diagnosing and testing HIV and other diseases. Community-based or facility-based testing programs could incorporate this approach in practice to identify high-risk individuals. However, additional studies are needed to further optimize this model, integrate and apply them into a real-world primary care setting. This may also disclose individuals who may need PrEP, among other risk reduction strategies.

## Supplementary Information

**Additional file 1:**

## Data Availability

The datasets used and/or analysed during the current study are available from the corresponding author on reasonable request. The code used in the analysis is publicly available on Github (https://github.com/charlesmutai/solid-disco).

## Abbreviations

UNAIDS: The Joint United Nations Programme
HIV: Human Immunodeficiency Virus
AIDS: Acquired Immune Deficiency Syndrome
PHIA: Population-based HIV Impact Assessment
DHS: Demographic and Health Surveys
ART: Antiretroviral therapy
UNAIDS: The Joint United Nations Programme
PrEP: Pre-exposure prophylaxis
CDC: Centers for Disease Control and Prevention
PEPFAR: President's Emergency Plan for AIDS Relief
EN: Elastic Net
KNN: K-Nearest Neighbors
RF: RandomForest
SVM: Support Vector Machine
LGBT: Light Gradient Boosting
MICE: Multiple imputations with chained equations
SFFS: Sequential forward floating selection
SHAP: SHapley Additive exPlanations

## References

[1] ‘UNAIDS data 2020’. https://www.unaids.org/en/resources/documents/2020/unaids-data. Accessed 29 Oct 2020.

[2] ‘Zambia | UNAIDS’. https://www.unaids.org/en/regionscountries/countries/zambia. Accessed 10 Nov 2020.

[3] ‘Fast-track commitments to end AIDS by 2030 | UNAIDS’. https://www.unaids.org/en/resources/documents/2016/fast-track-commitments. Accessed 30 Nov 2020.

[4] ‘2016 United Nations Political Declaration on Ending AIDS sets world on the Fast-Track to end the epidemic by 2030’. https://www.unaids.org/en/resources/presscentre/pressreleaseandstatementarchive/2016/june/20160608_PS_HLM_PoliticalDeclaration. Accessed 29 Oct 2020.

[5] Jewell BL (2020). Potential effects of disruption to HIV programmes in sub-Saharan Africa caused by COVID-19: results from multiple mathematical models. Lancet HIV.

[6] Dorward J (2021). The impact of the COVID-19 lockdown on HIV care in 65 South African primary care clinics: an interrupted time series analysis. Lancet HIV.

[7] Cuadros DF, et al., ‘Mapping the spatial variability of HIV infection in Sub-Saharan Africa: Effective information for localized HIV prevention and control’. Sci Rep. 7, 1, 1, 2017, 10.1038/s41598-017-09464-y. 10.1038/s41598-017-09464-y PMC5567213 28831171

[8] Zulu LC, Kalipeni E, Johannes E, Analyzing spatial clustering and the spatiotemporal nature and trends of HIV/AIDS prevalence using GIS: the case of Malawi, 1994–2010. BMC Infect Dis. 2014;14(1):285. 10.1186/1471-2334-14-285. 10.1186/1471-2334-14-285 PMC4057596 24886573

[9] Huerga H, et al. Who Needs to Be Targeted for HIV Testing and Treatment in KwaZulu-Natal? Results From a Population-Based Survey’, J. Acquir. Immune Defic Syndr. 2016;73(4):411–8. 10.1097/QAI.0000000000001081. 10.1097/QAI.0000000000001081 PMC5172512 27243903

[10] Blower S, Coburn BJ. Maximising the effect of combination HIV prevention in Kenya. Lancet Lond Engl. 2014;384(9952):1426. 10.1016/S0140-6736(14)61859-6. 10.1016/S0140-6736(14)61859-6 25390322

[11] Aral SO, Torrone E, Bernstein K (2015). Geographical targeting to improve progression through the sexually transmitted infection/HIV treatment continua in different populations. Curr Opin HIV AIDS.

[12] ‘Social and Behavioural Aspects of the HIV Epidemic--A Review on JSTOR’. https://www.jstor.org/stable/2982186. Accessed 6 Nov 2020.

[13] Rizza SA, MacGowan RJ, Purcell DW, Branson BM, Temesgen Z (2012). HIV Screening in the Health Care Setting: Status, Barriers, and Potential Solutions. Mayo Clin Proc.

[14] Celum C, Barnabas R (2019). Reaching the 90–90-90 target: lessons from HIV self-testing. Lancet HIV.

[15] Zheng W, Balzer L, van der Laan M, Petersen M (2018). Constrained binary classification using ensemble learning: an application to cost-efficient targeted PrEP strategies. Stat Med.

[16] Obermeyer CM, Osborn M (2007). The utilization of testing and counseling for HIV: a review of the social and behavioral evidence. Am J Public Health.

[17] De Cock KM, Barker JL, Baggaley R, El Sadr WM. Where are the positives? HIV testing in sub-Saharan Africa in the era of test and treat’, AIDS Lond Engl. 2019;33(2):349–52. 10.1097/QAD.0000000000002096. 10.1097/QAD.0000000000002096 30557162

[18] Ahmed S (2016). Lost opportunities to identify and treat HIV-positive patients: results from a baseline assessment of provider-initiated HIV testing and counselling (PITC) in Malawi. Trop Med Int Health.

[19] J. Sidey-Gibbons and C. Sidey-Gibbons, ‘Machine learning in medicine: a practical introduction’, BMC Med Res Methodol. 2019;19. 10.1186/s12874-019-0681-4. 10.1186/s12874-019-0681-4 PMC6425557 30890124

[20] Tang D, et al., ‘Application of Data Mining Technology on Surveillance Report Data of HIV/AIDS High-Risk Group in Urumqi from 2009 to 2015’, Complexity, 10, 2018. https://www.hindawi.com/journals/complexity/2018/9193248/. Accessed 17 Feb 2021.

[21] Hailu T (2015). Comparing Data Mining Techniques in HIV Testing Prediction. Intell Inf Manag.

[22] Agrebi S, Larbi A. Use of artificial intelligence in infectious diseases. Artif Intell Precis Health. 2020:415–38. 10.1016/B978-0-12-817133-2.00018-5.

[23] Marcus JL, Sewell WC, Balzer LB, Krakower DS (2020). Artificial Intelligence and Machine Learning for HIV Prevention: Emerging Approaches to Ending the Epidemic. Curr HIV/AIDS Rep.

[24] Klon AE, Glick M, Davies JW (2004). Application of machine learning to improve the results of high-throughput docking against the HIV-1 protease. J Chem Inf Comput Sci.

[25] Marcus JL, Hurley LB, Krakower DS, Alexeeff S, Silverberg MJ, Volk JE (2019). Use of electronic health record data and machine learning to identify candidates for HIV pre-exposure prophylaxis: a modelling study. Lancet HIV.

[26] A. I. L. LearningNeuroscienceNeurotech·July 8 and 2019, ‘Two new algorithms can identify patients at risk of HIV’. Neuroscience News. 2019. https://neurosciencenews.com/hiv-algorithms-14441/. Accessed 2 Nov 2020.

[27] Kumari S, Chouhan U, Suryawanshi SK. ‘Machine learning approaches to study HIV / AIDS infection: A Review’, 2012. 10.21786/bbrc/10.1/6.

[28] Lee JS, Paintsil E, Gopalakrishnan V, Ghebremichael M. ‘A comparison of machine learning techniques for classification of HIV patients with antiretroviral therapy-induced mitochondrial toxicity from those without mitochondrialtoxicity. BMC Med Res Methodol. 2019;19(1):216. 10.1186/s12874-019-0848-z. 10.1186/s12874-019-0848-z PMC6882363 31775643

[29] Orel E, Esra R, Estill J, Marchand-Maillet S, Merzouki A, Keiser O. ‘Machine learning to identify socio-behavioural predictors of HIV positivity in East and Southern Africa’, medRxiv. 2020. 10.1101/2020.01.27.20018242.

[30] ‘PHIA Project Document Manager - Datasets’. https://phia-data.icap.columbia.edu/files. Accessed 29 Oct 2020.

[31] ‘Population-based HIV Impact Assessment (PHIA) Data Use Manual ​ - Google Search’. https://www.google.com/search?q=Population-based+HIV+Impact+Assessment+(PHIA)+Data+Use+Manual+%E2%80%8B&oq=Population-based+HIV+Impact+Assessment+(PHIA)+Data+Use+Manual+%E2%80%8B&aqs=chrome..69i57.585j0j7&client=ubuntu&sourceid=chrome&ie=UTF-8. Accessed 29 Oct 2020.

[32] Kuhn M, Johnson K. Feature Engineering and Selection: A Practical Approach for Predictive Models, 1st edition. Chapman and Hall/CRC, 2019.

[33] Buuren S, Groothuis-Oudshoorn C. ‘MICE: Multivariate Imputation by Chained Equations in R. J Stat Softw. 2011;45. 10.18637/jss.v045.i03.

[34] Prashanth DS, Mehta RVK, Sharma N (2020). Classification of Handwritten Devanagari Number – An analysis of Pattern Recognition Tool using Neural Network and CNN. Procedia Comput Sci.

[35] Zou H, Hastie T. ‘Regularization and variable selection via the elastic net’, J R Stat Soc Ser B Stat Methodol. 2005;67(2):301–20. 10.1111/j.1467-9868.2005.00503.x.

[36] Zhang Z (2016). Introduction to machine learning: k-nearest neighbors. Ann Transl Med.

[37] Breiman L (2001). Random Forests. Mach Learn.

[38] Cortes C, Vapnik V (1995). Support-vector networks. Mach Learn.

[39] Chen T, Guestrin C. ‘Xgboost: A scalable tree boosting system’, in Proceedings of the 22nd acm sigkdd international conference on knowledge discovery and data mining, 2016, 785–794.

[40] Fan J, Ma X, Wu L, Zhang F, Yu X, Zeng W. Light Gradient Boosting Machine: An efficient soft computing model for estimating daily reference evapotranspiration with local and external meteorological data. Agric Water Manag. 2019;225.

[41] ‘F-Score’, DeepAI, May 17, 2019. https://deepai.org/machine-learning-glossary-and-terms/f-score. Accessed 20 May 2021.

[42] Goutte C, Gaussier E (2005). ‘A Probabilistic Interpretation of Precision. Recall and F-Score, with Implication for Evaluation’.

[43] Saito T, Rehmsmeier M. ‘The Precision-Recall Plot Is More Informative than the ROC Plot When Evaluating Binary Classifiers on Imbalanced Datasets’, PLOS ONE. 2015;10(3):e0118432. 10.1371/journal.pone.0118432. 10.1371/journal.pone.0118432 PMC4349800 25738806

[44] ‘A Unified Approach to Interpreting Model Predictions’. https://papers.nips.cc/paper/7062-a-unified-approach-to-interpreting-model-predictions. Accessed 2 Nov 2020.

[45] Agot KE, Ndinya-Achola JO, Kreiss JK, Weiss NS (2004). Risk of HIV-1 in Rural Kenya: A Comparison of Circumcised and Uncircumcised Men. Epidemiology.

[46] Baranczuk Z, et al., ‘Socio-behavioural characteristics and HIV: findings from a graphical modelling analysis of 29 sub-Saharan African countries’, J. Int. AIDS Soc. 2019;22(12):e25437. 10.1002/jia2.25437. 10.1002/jia2.25437 PMC6921084 31854506

[47] Sing RK, Patra S. ‘What Factors are Responsible for Higher Prevalence of HIV Infection among Urban Women than Rural Women in Tanzania?’, Ethiop. J Health Sci. 2015;25(4):4. 10.4314/ejhs.v25i4.5. 10.4314/ejhs.v25i4.5 PMC4762970 26949296

[48] Kharsany ABM, Karim QA (2016). HIV Infection and AIDS in Sub-Saharan Africa: Current Status, Challenges and Opportunities. Open AIDS J.

[49] Mondal MNI, Shitan M. ‘Factors affecting the HIV/AIDS epidemic: An ecological analysis of global data. Afr Health Sci. 2013;13(2):2. 10.4314/ahs.v13i2.15. 10.4314/ahs.v13i2.15 PMC3824495 24235928

[50] Suthar AB, et al., ‘Towards Universal Voluntary HIV Testing and Counselling: A Systematic Review and Meta-Analysis of Community-Based Approaches’. PLOS Med. 2013;10(8):e1001496. 10.1371/journal.pmed.1001496. 10.1371/journal.pmed.1001496 PMC3742447 23966838

[51] Dellar RC, Dlamini S, Karim QA. ‘Adolescent girls and young women: key populations for HIV epidemic control. J Int AIDS Soc. 2015;18(2 Suppl 1):19408. 10.7448/IAS.18.2.19408. 10.7448/IAS.18.2.19408 PMC4344544 25724504

[52] ‘Women and girls, HIV and AIDS’, Avert, Jul. 20, 2015. https://www.avert.org/professionals/hiv-social-issues/key-affected-populations/women. Accessed 13 Nov 2020.

[53] Yazdanpanah Y, et al. Routine HIV Screening in France: Clinical Impact and Cost-Effectiveness. PLoS ONE. 2010;5. 10.1371/journal.pone.0013132 PMC2956760 20976112

[54] Sullivan A, et al. Feasibility and Effectiveness of Indicator Condition-Guided Testing for HIV: Results from HIDES I (HIV Indicator Diseases across Europe Study). PLoS ONE. 2013;8. 10.1371/journal.pone.0052845 PMC3546115 23341910

[55] ‘WHO | WHO expands recommendation on oral pre-exposure prophylaxis of HIV infection (PrEP)’, WHO. http://www.who.int/hiv/pub/prep/policy-brief-prep-2015/en . Accessed 24 May 2021.

[56] Kagaayi J, et al., ‘Indices to measure risk of HIV acquisition in Rakai, Uganda’, PloS One. 2014;9(4):e92015. 10.1371/journal.pone.0092015. 10.1371/journal.pone.0092015 PMC3976261 24704778

[57] Cambiano V, Miners A, Phillips A (2016). What do we know about the cost-effectiveness of HIV preexposure prophylaxis, and is it affordable?. Curr Opin HIV AIDS.

[58] Maughan-Brown B, Venkataramani AS. ‘Accuracy and determinants of perceived HIV risk among young women in Africa. BMC Public Health. 2017;18. 10.1186/s12889-017-4593-0. 10.1186/s12889-017-4593-0 PMC5520344 28732496

[59] ‘WHO | Guideline on when to start antiretroviral therapy and on pre-exposure prophylaxis for HIV’. https://www.who.int/hiv/pub/guidelines/earlyrelease-arv/en/. Accessed 24 May 2021. 26598776

