# Supplementary material for: Use of machine learning techniques to identify HIV predictors for screening in sub-Saharan Africa
Source: BMC Med Res Methodol. 2021 Jul 31;21:159. doi: 10.1186/s12874-021-01346-2 (PMC8325403; doi:10.1186/s12874-021-01346-2)
Supplement: Supplementary file 1 — Additional file 1: [file 12874_2021_1346_MOESM1_ESM.docx]

## **Supplementary Material**

**Table A1. Variables excluded in pre-processing**

**============ ======================== ==================**

**Pre-processing Females (No. of variables) Males (No. of variables)**

**============ ======================= ================**

Total available variables 238 238

More than 30% missing 124 128

^^[[1]](#footnote-2)^^Non-unique columns 6 19

^^[[2]](#footnote-3)^^Duplicate columns 5 5

^^[[3]](#footnote-4)^^Non-informative 46 42

Above 0.8 correlated features 21 18

Total excluded 202 212

=============== ========== ============ =========== ==========

**Table A2. PHIA summary of the features used the analysis**

| **Features** | **Female** | **Male** | **Category** |
| --- | --- | --- | --- |
| Age | √ | √ | 15 to 80 years |
| Relationship with the family head | √ | √ | Head, Wife/Husband/Partner, Son or Daughter, Son-in-law/Daughter-in-law, Grandchild, Parent, Parent-in-law, Brother/Sister, Co-wife, Other Relative, Adopted/Foster/Stepchild, Not related |
| Respondent live in household' | √ | √ | Yes, No |
| Sick to work in the last three months | √ | √ | Yes, No |
| Ever attended school | √ | √ | Yes, No |
| Ever enrolled in school | √ | √ | Yes, No |
| Highest level of education | √ | √ | Pre-primary, Primary, Post-primary training, Secondary (O-Level), Post-secondary (O-Level) Training, Secondary(A-Level), Post-secondary-Level) Training, University |
| Highest grade at that school level | √ | √ |  |
| Work for payment in the last 12 months | √ | √ | Yes, No |
| Ever married/lived together | √ | √ | Yes, No |
| Avoiding pregnancy | √ | √ | Yes, No |
| Age at first sex | √ | √ | 8 to 56 years |
| Ever tested HIV | √ | √ | Ever tested, Never tested |
| Ever sought TB treatment | √ | √ | Yes, No |
| Alcohol drink frequency | √ | √ | Never, once a month or less, 2 to 4 times a month,  2 to 3 times a week, 4 or more times a week |
| Urban area indicator | √ | √ | Urban, Rural |
| Known HIV status | √ | √ | Stated Positive, Stated Negative, Never Tested, Don’t |
| Wealth quintile | √ | √ | Lowest, Second, Middle, Fourth, Highest |
| HIV status | √ | √ | Positive, Negative |
| Had sexual intercourse past 12 months | √ | √ | Yes, No |
| Ever had sexual intercourse | √ | √ | Yes, No |
| Country | √ | √ | Tanzania, Zambia, Malawi, Eswatini |
| Marital status | √ | √ | Married, Living together, Widowed, Divorced, Separated |
| No. of times pregnant | √ |  | 0 to 23 times |
| No. of births since 2012 | √ |  | 0 to 10 children |
| Ever had a successful birth | √ |  | Yes, No |
| Whether pregnant now | √ |  | Yes, No |
| Circumcision status |  | √ | Yes, No |
| No. of children given birth since 2012 | √ |  |  |
| Tested HIV at pregnancy | √ |  | Yes, No |
| Delivered 12 months preceding survey | √ |  | Yes, No |
| Self-reported ARV status during pregnancy | √ |  | Yes, No |
| Breastfed last child | √ |  | Yes, No |
| Bought/sold sex in the past 12 months | √ | √ | Yes, No |
| Condom was used at last paid sex in past 12 months | √ | √ | Yes, No |
| Last sex partner relations past 12 months | √ | √ | Spouse,sex-worker, friend, stranger,ex-partner |
| LAg: recent/long term infection | √ |  | Positive: recent, Positive: long term |

**Table A3. Background characteristics of the PHIA dataset**

|  |  | **Female** | | **Male** | |
| --- | --- | --- | --- | --- | --- |
| **Characteristics** | **Levels** | **HIV Negative** | **HIV Positive** | **HIV**  **Negative** | **HIV**  **Positive** |
| Total number of individual |  | 39124 | 5981 | 38387 | 3552 |
| Country (n, % of total) | Malawi | 2025 (34.1) | 590 (46.1) | 2053 (37.3) | 506 (43.4) |
|  | Eswatini | 1715 (28.9) | 328 (25.6) | 1460 (26.5) | 342 (29.3) |
|  | Tanzania | 1353 (22.8) | 245 (19.1) | 1253 (22.8) | 223 (19.1) |
|  | Zambia | 842 (14.2) | 117 (9.1) | 739 (13.4) | 95 (8.2) |
| Current age ( n, % of total) | 15-19 | 438 (0.6) | 26 (0.2) | 373 (0.5) | 29 (0.5) |
|  | 20-24 | 359 (0.5) | 16 (0.1) | 252 (0.4) | 18 (0.3) |
|  | 25-29 | 252 (0.3) | 3 (0.0) | 208 (0.3) | 12 (0.2) |
|  | 30-34 | 266 (0.3) | 1 (0.0) | 250 (0.4) | 2 (0.0) |
|  | 35-39 | 8751 (12.1) | 245 (2.3) | 8569 (12.0) | 101 (1.7) |
|  | 40-44 | 7079 (9.8) | 541 (5.1) | 7107 (10.0) | 155 (2.6) |
|  | 45-49 | 5506 (7.6) | 920 (8.6) | 5601 (7.9) | 319 (5.4) |
|  | 50-54 | 4524 (6.3) | 1074 (10.1) | 4402 (6.2) | 515 (8.7) |
|  | 55-59 | 3354 (4.6) | 1049 (9.8) | 3425 (4.8) | 615 (10.4) |
|  | 60-64 | 2660 (3.8) | 826 (7.7) | 2695 (3.8) | 620 (10.5) |
|  | 65-69 | 8981 (12.4) | 1261 (11.8) | 8748 (12.3) | 839 (14.1) |
|  | 70-74 | 4314 (6.0) | 2079 (19.5) | 4331 (6.1) | 1151 (19.4) |
|  | 75-79 | 9414 (13.0) | 1580 (14.8) | 9297 (13.0) | 989 (16.7) |
|  | 80+ years | 16415 (22.7) | 1061 (9.9) | 16011 (22.5) | 573 (9.6) |
| Type of residence, n (% of total) | Urban | 7289 (48.8) | 989 (51.1) | 6697 (47.0) | 612 (49.2) |
|  | Rural | 7633 (51.2) | 948 (48.9) | 7556 (53.0) | 631 (50.8) |
| Wealth quintile, n (% of total) | Lowest | 7963 (12.6) | 1331 (13.3) | 7983 (12.8) | 733 (12.5) |
|  | Second | 7624 (12.1) | 1383 (13.8) | 7757 (12.4) | 801 (13.6) |
|  | Middle | 8555 (13.5) | 1320 (13.2) | 8338 (13.3) | 769 (13.1) |
|  | Fourth | 26146 (41.3) | 3571 (35.7) | 25859 (41.4) | 2225 (38.0) |
|  | Highest | 12978 (20.5) | 2410 (24.0) | 12528 (20.1) | 1327 (26.6) |
| Highest level of school, n (% of total) | Primary | 23406 (59.9) | 20587 (61.4) | 19559 (54.9) | 1678 (51.0) |
|  | Post primary | 8635 (22.1) | 6978 (20.8) | 8752 (24.5) | 1011 (30.7) |
|  | Secondary (O-level) | 5717 (14.6) | 5028 (15.0) | 5876 (16.5) | 518 (15.7) |
|  | Post-Secondary | 1097 (2.8) | 945 (2.8) | 971 (2.7) | 78 (2.4) |
|  | Secondary (A-level) | 214 (97.7) | 5 (0.0) | 509 (1.4) | 6 (0.2) |


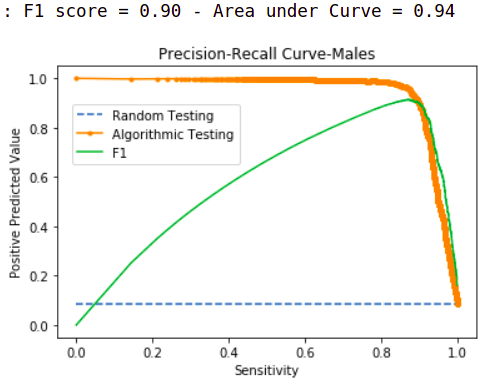
Figure A1: Precision-Recall Curves for the XGBoost model in males


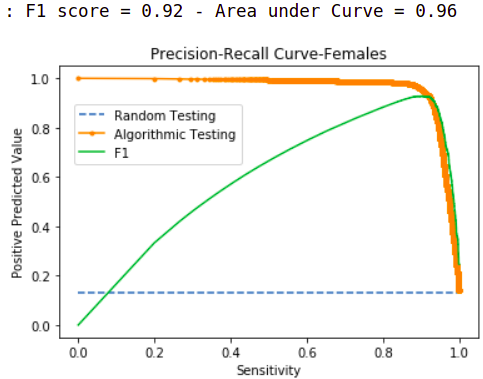
Figure A2: Precision-Recall Curves for the XGBoost model in females

1. Variables that have different names but the same attributes. [↑](#footnote-ref-2)
2. Variables in the dataset that are repeated. [↑](#footnote-ref-3)
3. Features such as.household-id, person-id, line-number, and others are non-informative on HIV. [↑](#footnote-ref-4)
